# The educational paradigm shift—a phenomenographic study of medical teachers’ experiences of practices

**DOI:** 10.1186/s12909-023-04013-w

**Published:** 2023-01-14

**Authors:** Sanna Brauer, Jaana Kettunen, Anna Levy, Jussi Merenmies, Petri Kulmala

**Affiliations:** 1 Faculty of Medicine, University of Oulu, Oulu, Finland; 2 Finnish Institute for Educational Research, University of Jyväskylä, Jyväskylä, Finland; 3 University of Helsinki, Helsinki University Hospital, Helsinki, Finland

**Keywords:** Undergraduate medical education, Medical teacher, Phenomenography, Competence orientation, Curriculum

## Abstract

**Background:**

This paper proposes a novel approach to the development of competence-oriented higher education, a national transformation aimed at harmonising and digitising undergraduate medical and dental education in Finland.

**Methods:**

We apply phenomenography as a viable qualitative method for medical education research. To better understand medical teachers’ expectations towards the change in the educational paradigm, we need to study teachers’ experiences of the current practices in undergraduate medical and dental education. The phenomenographic approach facilitates solid links between research, educational development, and change.

**Results:**

The phenomenographic study maps the qualitatively different ways in which medical teachers experience undergraduate medical and dental education practices. The answers reflect the changing educational paradigm in medical schools, suggesting practical implications for further development of medical and dental education and training. Core content analysis is preferred instructional scaffold for both teachers and students to prioritise the extensive medical education objectives. The change towards competence-based orientation is in progress and national co-operation accelerates its impact.

**Conclusion:**

There is an obvious need to enrich the content of the current curriculum with national guidelines that aim for congruence in assessment and objectives. Our results suggest an assessment application for the theoretical concepts presented and promote the competence orientation of education throughout the curricula of medical and dental undergraduate education. Moreover, our results contribute to current European discourses on competence-based approaches in higher education. Up-to-date pedagogical faculty development programmes are a key prerequisite for teacher empowerment and future orientation in teaching and learning for healthcare professions.

## Introduction

The key factor in ensuring and enhancing the quality of health systems is high-level education that transforms learners into qualified health professionals [1, 2]. A competence-based approach to higher education (HE) has grown in recent years and the European Union promotes the provision of competence-oriented education, training, and learning [3–5]; however, the notion of competence includes a variety of attributes, and competence-based models are widely considered to be discipline- and organisation-specific [4]. As Ottenhoff-de Jonge et al. [6] point out, the educational beliefs of medical educators drive their actions while teaching. In general, these unique views on teacherhood are socially constructed and contain both cognitive and affective components [6]. Furthermore, reconceptualising teacherhood is important for medical and dental schools to improve the quality of education and to lead the change from teaching-centred practices to a competence-based orientation with the learner at the centre [6, 7]. In addition, this profound paradigm shift requires re-thinking regarding the faculty development of medical educators. A standardisation of practices is required to avoid inconsistent developmental approaches, which could ultimately affect the quality of health care.

In medical education different frameworks serve various roles outlining characteristics of a competent workforce, facilitating mobility, and developing or assessing professional expertise [7]. Advanced medical knowledge based on basic science is essential for a physician, but in clinical practice they are often assessed based on non-medical expertise, such as collaboration and communication [8]. This study focuses on medical teachers’ different perceptions and sometimes tacit expectations of successful undergraduate medical and dental education.

Widely employed frameworks such as CanMEDs [9] and the British national preferences of “Outcomes for Graduates” [10] are able to describe the abilities physicians are required to have to meet evolving healthcare needs. CanMEDs [9] is a Canadian competency-based framework for medical training that has been widely adopted in specialty training and increasingly in undergraduate medical education around the globe. The competencies of CanMEDs [8] are integrated into postgraduate medical education [8] in Finland, and therefore this framework is worthy of attention in this article. CanMEDs [9] covers a broad perspective of the competence continuum of a physician needed to meet societal needs in an increasingly demanding and evolving health care sector. Frameworks have been recognised in transforming Canadian medical training since the 1990s as valid and practical foundations for excellence in patient care. The aim of the comprehensive CanMEDs framework is to facilitate acquiring up-to-date and sustainable expertise from entering residency programmes to transitions to autonomous practice as well as the continuous development of professional competence.

Currently, there are two prominent approaches to competence assessment in medical education: “an analytic approach that aims to precisely measure observable constituents and facets of competence and a holistic approach that focuses on a comprehensive assessment of competences in complex real situations reflecting actual performance” [11]. Nevertheless, the construction of competence frameworks for healthcare professions is considered challenging due to “potentially inadequate descriptions of practice, variable developmental approaches, and inconsistent reporting and evaluating of outcomes” [8] (p1355). The developmental efforts in HE represent a wide variation in approaches to competence, complicating the achievement of coordinated policies [4]. All domains of medical education must prepare graduates to meet the requirements of the emerging themes, such as patient care and safety [9]. The need for standardised development is obvious.

### Comprehensive development towards competence orientation

Traditionally, medical, and dental education programmes have been based on the time spent on training [8]. In competence-based medical education, the capabilities of a graduating student are defined in advance. The extensive adoption of a competence-based approach to medical education aims to integrate theory with clinical practice supporting competence development as a continuum [11–13]. The requirements for training even more versatile and qualified physicians and dentists are increasing, and therefore a strong investment is needed to develop the professional skills of medical and dental teachers. The competence-based orientation may seem a rather theoretical and firmly pre-structured process for an experienced teacher [8], and the concepts and practices may be unfamiliar to teachers with a strong academic background [5]; however, the purpose is to adopt practices to support the continuum of professional development throughout students’ studies and working careers. This significant paradigm shift is a key challenge in Finland.

In medical education development has recently focused on identifying and forming the core curricula. The intended curriculum is the part of the curriculum that supports educational objectives and is planned by a curriculum committee [14]. What students actually learn (the learned curriculum) differs from the intended curriculum based on a core content analysis[15]. Moercke and Eika found that “the learned curriculum of clinical skills constituted 75% of the intended curriculum” [14] (p477). Another aspect to the realisation of the intended curriculum is how teachers interpret and implement the given guidelines in the classroom, constituting a taught/enacted curriculum [14, 15]. The assessed curriculum allows the impact of planned and enacted curricula to be evaluated, revealing students’ achievements and acquired competences [15].

Entrustable Professional Activities (EPAs) refer to the tasks in a professional setting that may be delegated to newly qualified doctors [9]. EPAs promote assessments in authentic settings that require a demonstration of competence in the daily practices of a physician [9]. The transition to competence-based specialist training has also prompted the reform of undergraduate education [8, 16]. Bramley and McKenna [16] report EPAs as a major development in identifying skill gaps in individuals or student cohorts and facilitating improvements. A key responsibility of students is to possess the appropriate level of competence before entering their residency programmes [8, 9]. This outcome-based approach to medical education aims to promote communication, collaboration, and management skills as key competences in addition to advanced medical expertise [8]. These qualities are extremely important to clearly communicate medical information to patients. Cooperation and collaboration are particularly important competences in the daily practice of other health care personnel as well [8].

EPAs are also employed in the evaluation of diverse curriculum models and in identifying curriculum gaps [16]. Ten Cate et al. [17] discuss the practical implications of EPAs for curricular work, which exist alongside their impact on the development of assessment practices and learning environments. According to Rotthoff et al. [11] “the concept of EPAs represents a paradigm between standardization and authenticity or control and trust in competence-based assessment.” They suggest using the “Competence Assessment Continuum Approach” to reorganize and adapt competence assessment. In addition to EPAs, Englander et al. [18] believe curricular development employs milestones and competence metrics describing individual competency. They define “a milestone as an observable marker of an individual’s ability that expresses the stepwise progression of expertise and EPA as a professional task that requires sufficient knowledge, skills, and attitudes and leads to a work outcome” [18] (p582).

Literature is replete with studies [19] indicating a relationship between approaches to teaching and the quality of students’ learning outcomes. Both students’ and teachers’ earlier experiences and perceptions inform the adoption of in-depth approaches in different contexts [19, 20]. Practicing medicine in a work context during the studies highlights potential skills gaps for undergraduates, but the lack of assessment tools makes evaluation scattered [8]. Evans et al. emphasise self-assessment as “a fundamental skill that should be introduced into both undergraduate and postgraduate education” [21] (p513). The ability to assess one's own work critically is often declared a goal of higher education but is seldom explicitly indicated in the curriculum [21, 22]. Still, doctors are expected to regularly assess individual performance, set targets and goals for professional growth, and act accordingly to maintain and continuously develop competence [21, 22]. As a career progresses as a teacher, valid self-assessment becomes even more important. Detailed criteria and performance-based feedback should enhance accuracy in self-assessment of competence [22], as success is measured as advancements in the standard of practice [21].

The multifaceted development of medical education and related educational research efforts serve important societal purposes. Interest in medical education research has grown in recent decades [23, 24]. It should be noted that medical education research is contextual by nature [25]. A paradigmatic change requires the reconstruction of teacherhood and systematic research in medical education, both in classrooms and clinical practice. Moreover, medical teachers should be able to examine their own work and develop teaching and learning research based on it [25, 26]. In addition, attention should be paid to study methods in undergraduate medical education research [23, 27].

### Research question

The key research question is as follows: *What are medical teachers´ ways of experiencing the undergraduate medical and dental education practices in Finland?* The aim is to discover and describe the qualitative variation in teachers’ ways of experiencing the phenomenon.

## Methods

This study employed a phenomenographic approach e.g. [28–30]. The purpose of phenomenographic research is to explore how people, whether students, teachers, professionals or laymen, understand or experience phenomena and concepts [31]. The core aim is to capture the variations in different conceptions or experiences and the relationships between them. Central to phenomenography is its non-dualist ontological perspective in which persons and the world are considered inseparable [32, 33]. A conception, or a way of experiencing a specific phenomenon, is perceived as a relation between the person and the phenomenon. People’s different ways of conceptualising or experiencing the same phenomenon are viewed as internally related, representing different meanings concerning the phenomenon in question [28, 33]. Thus, the object of phenomenographic research is the phenomenon as experienced by individuals rather than the phenomenon itself.

Phenomenography is grounded on the premise that there is a limited number of ways of experiencing a particular phenomenon and that they are logically related. The primary outcome of phenomenographic analysis is a structured set of logically related categories describing qualitative variations in ways of experiencing or understanding the phenomenon in question at a collective level [34]. Phenomenography is a viable qualitative approach to educational research in HE [27]. It fits within the interpretivism paradigm, which acknowledges that there are multiple, diverse interpretations of reality [27].

### Study context and participants

The context of this study is Finnish undergraduate medical and dental education offered by five universities. In 2018, the intake of new students was 879, and the number of working age physicians in Finland [35] was 21,148. Basic medical education lasts for six years and confers a licentiate degree in medicine (360 ECTS credits), and dental programmes last five and a half years (330 ECTS credits). The basis for the educational and practice standards developing undergraduate medical education in Finland was recently updated [36]. The competence objectives are divided into three main categories: professional values and activities, professional skills, and professional knowledge. Competence objectives in dental education are based on the Association for Dental Education in Europe (ADEE) competence objectives. The teaching of clinical skills is integrated into all activities of the discipline-specific clinical period (three to six academic years). Students participate in the work of various hospital departments and health centres, where they learn the necessary medical skills. All medical schools have research programmes for students who wish to undertake scientific work. Student-centred learning methods, such as problem-based learning (PBL), have been introduced [37]. The current development efforts emphasise a competence orientation in medical education and national, discipline-specific collaboration in reaching a consensus on the core content of undergraduate education in medicine and dentistry [38].

The study participants were Finnish undergraduate medical and dental education teachers (*n* = 17; 6 men, 11 women) with medical degrees with different specialisations (*n* = 11), ranging in age group 31–35 years (*n* = *1*) to age group of 61–65 years (*n* = 4). Participants represented various employment backgrounds as medical doctors and as teachers. One was a specialising dentist, and the rest of the doctors were licensed medical specialists, eight specialised in dentistry. Their experience as a graduated physician or dentist ranged from less than 3 (*n* = 1) to more than 30 years (*n* = 7) and experience as a teacher from less than 3 years (*n* = 1) to more than 20 years (*n* = 1). Six participants had university pedagogical studies (10 ECTS or more), and one of them had teacher’s pedagogical qualifications (60 ECTS) in professional teacher education. Three participants had completed Special Competence Programme in Medical/Dental Education (115 h), which the Finnish Medical Association recognises. Almost half (*n* = 8) of the respondents lacked the teacher training. The majority of the respondents (11) had participated in some pedagogical short-term training during their careers, however only six of the teachers were involved in continuous pedagogical training within the last three years. One participant didn’t have any pedagogical training.

### Data collection

The data were collected in 2020 from three group interviews [39]. Interviews are the most common method used to obtain phenomenographic data, although there are other methods used [30, 40, 41]. Because the aim of this study was to investigate the range of different ways of experiencing the same phenomenon, we considered conducting group interviews an effective method to encourage the participants to express their thoughts, views, and experiences by interacting with one another. Furthermore, we considered group interviews appropriate because the aim of phenomenographic research is to capture collective rather than individual accounts of people’s experiences of a phenomenon. One group interview was conducted in the spring of 2020, and two online group interviews were conducted in the fall of 2020. Interviews were transcribed verbatim. The transcription of 217 min of interviews provided 80 pages for analysis.

Although group dynamics provide advantages during data collection, challenges may also arise, including an expectation of consensus rather than diversity, for which stronger members take the lead while others follow rather than expressing their own opinions and understandings [42]. While a facilitator may be able to mitigate this pressure to conform through clear instructions and the careful probing of the nuances of participants’ opinions as well as ensuring specific members do not dominate, it would be naive to expect that even excellent facilitation can remove all the pressures of social desirability [43]. In general, despite these limitations, the researcher plays a critical role in steering the conversation, involving the silent participants, and inviting participants to specify their views [44]. In the present study, smaller meetings were organised to promote equal opportunities to speak and to be heard. By having everyone participate from the beginning, the interviewer encouraged participants to speak and to feel confident in voicing their opinions. The interview questions were semi-structured [34]. The framework of themes to be explored were addressed by the following broadly structured questions:

How should students’ learning be supported in undergraduate medical and dental education?

What kind of pedagogical models have you personally applied? Which models do you know that are commonly used in medical and dental education?

What kind of pedagogical choices do you recognise as scaffolds for student learning?

What kind of guidance practices should be used to support learning?

How and what kind of learning environments support student learning?

What should the different tasks be like?

What kind of learning materials would be good to use?

How should the evaluation be carried out?

How does competence-based education fit into undergraduate education in medicine and dentistry?

What kind of pedagogical skills should a teacher possess for medical and dental education?

New ideas were discussed during the interviews by the participants, and the questions were asked as part of the discussion in the order in which the topics emerged. Follow-up questions or reformulated questions were asked when necessary to uncover underlying meanings [34]. Set of quantitative multiple-choice questions were used to map explanatory background variables, such as teaching experience and the field of specialisation.

### Data analysis

Data were analysed using a phenomenographic approach, following the guidelines and examples provided by Åkerlind [28], Marton [29, 30, 34] and Kettunen and Tynjälä [40]. The first phase of the analysis focused on identifying and describing participants’ ways of experiencing undergraduate medical and dental education practices in general terms. Transcripts were considered as a whole and were repeatedly re-read to obtain and identify the underlying foci and intentions expressed in them. No predetermined categories or theories were used, but they were constituted in relation to the data. A draft set of descriptive categories for collective meaning was developed by comparing and contrasting the identified similarities and differences in expressed sentiments.

The second phase of the analysis sought to delineate the logical relationships among the various categories. The aim was to reveal one way of seeing a phenomenon in comparison to another, more complex one [28, 30]. Common themes of variations in the participants’ ways of experiencing undergraduate medical and dental education practices were identified and used to structure the logical relationships both within and between the categories [28].

To ensure a robust analysis, the data were initially analysed by the first author, and a second opinion was then sought from research colleagues, with whom she met several times. Discussing and revising the categories and their structures in this way confirmed the valid interpretation of the data [40]. The final phase of the analysis focused on ensuring that the categories of description met the three quality criteria defined by Marton and Booth [30]: (a) each category describes a distinctly different way of experiencing the phenomenon; (b) a logical relationship between the different categories is hierarchically represented; and (c) a limited number of categories describing variation are presented.

## Results

Data analysis revealed four distinct categories of description reflecting participants’ ways of experiencing undergraduate medical and dental education practices in Finland (Table 1).

**Table 1:** Medical teachers’ ways of experiencing undergraduate medical and dental education practices in Finland

Dimension of Variation	Category 1: Established Situation	Category 2: Fragment Reassembly	Category 3: Accelerators of Change	Category 4: Future Orientation
*Competence Orientation*	Local	Regional	National	European
*Curriculum*	Intended	Interpreted	Co-Created	Assessed
*Teacher’s Role*	Experiment	Exemplify	Demonstrate	Reflect
*Teacherhood*	Insecurity	Appreciation of Competence	Empowerment	Continuous Development
*Learning Is Based on*	Repetition	Connection between Theory and Practice	Practical Applications	Interprofessional Interaction
*Achievement Goal*	Final Examination	Learning Objectives	Self-Assessment in Medical Practice	Professional Growth
*Instructional Scaffold*	Digital Learning Solutions	Core Content Analysis	Authentic Learning Opportunities	Assessment Criteria

### Description of the categories

In the first category, medical teachers experienced the undergraduate medical and dental education practices as an established situation to which they were accustomed. The competence orientation was based on *local* competence needs and related implications. The professional needs for competence were determined by teachers as individuals. Teaching was roughly based on the *intended* curriculum and the teacher’s role was to *experiment* with practices that were not defined in the official guidelines.

*Targeted learning outcomes are listed there [in the curriculum], but how they are actually implemented in teaching is up to the university.*

*The targeted learning outcomes guide teaching, but how they are achieved is for the teacher to decide.*

*[Teaching] has been focused on how [knowledge] is applied in practice.*

Participants expressed *insecurity* in teacherhood, and employed a variety of metrics to describe their credibility as teachers, no matter how experienced they were in the field of medicine itself. Learning was considered to be based on *repetition*, and the ultimate goal of the studies was the study module’s *final examination*. Medical teachers were familiar and confident with a variety of *digital learning solutions* that they considered essential instructional scaffolds.

*I don’t really know about these pedagogies.*

*Students simply learn through repetition.*

*They should graduate, and now they have this big block exam.*

*The digitization of everyday life can be seen in teaching and in work in the field.*

In the second category, medical teachers experienced the educational practices as fragmented requiring reassembly. The competence orientation was *regional*, reflecting multifaceted healthcare needs in particular areas, such as sparsely populated area of Lapland. Teachers deliberately *interpreted* the curriculum, referring to the actual curricular content they had been engaged with during their studies years ago.

*[In Northern Finland] we have to, for example, teach the graduating course, some of the same things that are part of the competences of an anaesthesiologist or medical specialist.*

*But, if I think of myself as a student, I think it would be easier and maybe better to [teach] one thing at a time.*

A teacher’s role was to *exemplify* how students will encounter the theoretical subject in actual patient work. Teacherhood was based on *appreciation of competence*, and expertise in pedagogy and medicine were genuinely respected. A teacher’s mission was to base learning on a *connection between theory and practice*.

*Even if there is research-based teaching, it should be well-linked to clinical, practical work, which is of course the highest level of competence.*

The competence to be achieved was described through *learning objectives* defined based on a *core content analysis*. *Core content analysis* was preferred instructional scaffold for both teachers and students to prioritise the extensive medical education objectives.

*It is probably easier for the student to concretely understand the targeted learning outcomes.*

*A core content analysis is a good tool for teachers and for curriculum work, evaluation and…planning teaching methods.*

*Students [use] the core content for exams in such a way that some only focus on those subjects because you should get through the exam if you know those things.*

The third category describes medical teachers' experiences of the educational practices as accelerators of change. Participants expressed that a *national* consensus on competence orientation is a crucial precondition for development. The curriculum was *co-created* and experienced as a change agent. The significance of nationwide collaboration and commitment to high-quality medical education were emphasised.

*Targeted learning outcomes and a core content analysis are done, and they are the same nationally, and we acknowledge that they exist.*

*The national [curricula] were created together by four universities.*

A teacher’s role was to *demonstrate* clinical practices in classroom teaching. Teacherhood was experienced as promoting professional *empowerment*. Learning was based on *practical applications*, and the goal was to equip students with competence to conduct *self-assessment in medical practice*. Instructional scaffolds to support this provided students with *authentic learning opportunities*.

*New things are also demonstrated through practice in teaching.*

*They are quite liberated when the teacher is like an equal peer in a certain way.*

*I got stuck reading those evaluations made by students about the emergency situation. Although I haven’t seen those situations at all, I claim that I almost know based on their answers what stage of competence development those students are already at, and I think this kind of self-reflection is important.*

*Systematic clinical work where the student can start small and then progress and develop and receive feedback on that. Then, when the skills have developed, they can do more. This process would naturally include the competence-based approach and targeted learning outcomes*.

The fourth category represents medical teachers' experiences of the educational practices through a future orientation. Competence orientation was growing in line with *European* guidelines and frameworks and curricular development efforts were based on an *assessed* curriculum. A teacher’s role was to *reflect* their own experiences and the diverse expectations of students, academia, and medical practice.

*General competence goals and assessment criteria are national and EU-based.*

*Curricula have been updated in the national level subject group.*

*The realization that has come to me is that basically, I start teaching them, the students, in the style in which I myself learn, and then you notice that not everyone learns well the way I learn.*

Teacherhood was believed to provide a decent chance of *continuous development*. Teachers who had participated in pedagogical training were interested in the new visions, pedagogical models, and tools. They were eager to apply what they learned immediately in their teaching. Learning was based on *interprofessional interaction* and seen in line with the requirement of *professional growth*.

*When I started studying University Pedagogy, I realized that our course is well-planned.*

*[We implement] simulations in a treatment room situation in such a way that there is a dental nurse, a patient, and a dentist, and they are role-played.*

*We should focus on what is essential and important in terms of working life and studying and growing up to be a physician.*

The instructional scaffolds that could have supported all previous efforts were *assessment criteria*. However, all forms, levels, and objectives of assessment were deemed to require considerable improvement.

*But, there is a need for development. I recognize that, for example, the assessment criteria are not transparent at all.*

*I would like more tools for the evaluation of clinical work so that the competences could be assessed.*

#### Relationships between the categories

Teachers’ experiences in undergraduate medical and dental education are shaped by many factors, such as the curriculum defined in official course guidelines, teaching practices, learning processes, and different phases of evaluation and assessment. In the first category, the educational practices were considered in relation to an *established situation* that offered little to no potential for change. The position of a teacher was not considered influential. The second category describes the fragmented educational practices and unique individual efforts to *reassemble fragments*. The third category describes the *accelerators of change* through current development efforts. The factors considered crucial for nation-level upheaval, harmonisation, and digitisation were collaboration and co-creation. The fourth category raises practical implications based on the current understanding of the future. Participants systematically expressed *future orientation* as the core value of curricula, and emphasised the essence of teachers’ wide-ranging pedagogical competence.

## Discussion

Phenomenographic results reveal different ways of experiencing undergraduate medical and dental education practices. The present situation does not appear to be optimal for the development of teaching and learning practices, given that teachers feel insecure about their pedagogical competence as they do not possess common metrics to describe their abilities or performance in a classroom. Moercke and Eika remind us that “educational objectives are often disseminated to teachers under the assumption that goals, learning objectives and checklists are agreed upon by developers, teachers and students alike” [14] (p477). Our findings are in line with their results: the method of implementing intended (planned) curricula seems to position teachers as passive co-operators and thus represents a top-down curriculum design process. Curriculum assessment would provide a sustainable way to evaluate the impact of planned and enacted curricula, as it reveals students’ achievements and acquired competences [15]. EPAs have the potential to be employed in the evaluation of diverse curriculum models or in identifying curriculum gaps [16]. The role of pedagogical experts in the process of defining the core curriculum needs to be discussed further.

In an established situation, aims and actions seem fragmented and a medical teacher’s ability to influence the prevailing conditions limited. Medical teachers have a strong sense of competence and a profound inner drive to review and improve current practices. Medical schools have well-established pedagogical practices worth developing into the digital era. Reactive strategies have been successful during COVID-19 pandemic [45, 46], and our results confirm that teachers are confident and comfortable facilitating already applied pedagogical choices online. However, they are not always able to rationalise their choices as their actions are based on individual experiences as a learner or teacher instead of conscious pedagogical consideration.

Our findings indicate that medical teachers are future orientated and have interest in developing teaching practices and the profession. They thrive as developers when collaboration and co-creation is supported. National interdisciplinary co-operation accelerates their impact. A lack of instructional scaffolds, such as assessment criteria, discourages many of these efforts, from planning to classroom actions. Core content analysis is perceived as an appropriate tool to allocate resources and focus actions as current medical education requires students to master extensive amounts of information. Biesta and van Braak [25] propose a triad of professional qualification, professional socialisation, and professional subjectification to reorient curriculum design, pedagogy, assessment, and evaluation in educational practice. Rotthoff et al. [11] criticise the assumption that the deconstruction of professional roles into variables, such as the trinity of knowledge, skills, and attitudes, automatically leads to comprehensive competences. Still, EPAs have been reported to improve student assessment and are well accepted in workplace assessment [16]. Ruhalahti [20] and Alkhowailed et al. [45] suggest rethinking assessment as an integral step in a learning process, with a deep-learning approach in mind. Our results confirm that practices of authentic assessment have not yet been established [45, 46]. Re-evaluation and empirical substantiation of competence-based assessment is required.

The results reflect doctors' perceptions of the changing educational paradigm in medical schools, suggesting practical implications for the further development of medical and dental education and training. Biesta and van Braak [25] emphasise that aims and actions should always be considered in relation to context. A competence-based approach should enhance accuracy in different forms of assessment as success is always measured as advancement in practice [21, 22]. This paradigmatic change requires the reconstruction of teacherhood and medical education research, both in classrooms and clinical practice. Our results emphasise that a medical teacher's role reflects the diverse expectations of students, academia, and medical practice. This reinforces the notion that systematic teacher research can improve the practice of education [25, 26]. Advanced medical knowledge is based on highest standards of medical science [12], and the latest medical education research should inform teachers’ aims and actions [25]. Future research could study additional educational practices in medical schools focusing on clinical lecturers’ pedagogical competence.

### Limitations and strengths

Phenomenographic studies help to improve practice by exploring variations in participants’ experiences of the phenomenon in question, which are illuminated by the dimensions of variation between them e.g. [28, 40]. The results of this study cannot be generalised, but they may be transferable to similar situations or applicable in another context.

The number of participants (*n* = 17) can be considered appropriate in light of previous phenomenographic studies suggesting that 10 to 15 participants is usually sufficient for capturing variation [27, 47, 48]. The sample was not completely random in that we selected active teachers as respondents. We cannot exclude the possibility that our findings could be an expression of other latent or unexplored dimensions related to the phenomenon.

## Conclusions

There is a need to enrich the content of the curriculum with national guidelines aiming at congruence between assessment and objectives. Our results suggest an assessment application of the theoretical concepts delivered and promote the competence orientation at different depths and breadths in medical and dental undergraduate education curricula. Development of assessment criteria is crucial. A holistic view of competence oriented HE should be emphasised. Medical education requires pan-European guidelines, national frameworks, and genuine sectorial/disciplinary collaboration on implementation. Up-to-date pedagogical faculty development programme [49] is a key prerequisite for teacher empowerment and a future orientation in teaching and learning for healthcare professions. Transforming educational beliefs and reconceptualising teacherhood in medical and dental education could increase the prestige of the teaching profession both at the level of society and within the scientific community. Concepts and indicators for medical teachers’ competence are required.

## Data Availability

Due to confidentiality agreements, supporting data can only be made available to bona fide researchers subject to a non-disclosure agreement. The anonymized datasets of the current study (in Finnish) are available from the corresponding author on reasonable request.
